# Treatment of proximal humerus fractures in geriatric patients - Can pathological DEXA results help to guide the indication for allograft augmentation?

**DOI:** 10.1371/journal.pone.0230789

**Published:** 2020-04-09

**Authors:** Sascha Halvachizadeh, Till Berk, Thomas Rauer, Christian Hierholzer, Roman Pfeifer, Hans-Christoph Pape, Florin Allemann

**Affiliations:** Department of Trauma, University Zurich, UniversitätsSpital Zürich, Zurich, Switzerland; Assiut University Faculty of Medicine, EGYPT

## Abstract

**Introduction:**

Reconstruction of proximal humerus fracture continues to represent a challenge, especially in severe osteopenia. However, there still is a lack of consensus and clear indication on use of allograft augmentation. Therefore, this study aims to investigate outcome after osteosynthesis with and without allograft augmentation. It focuses on bone density results obtained by DEXA as potential examination that might help decision-making.

**Methods:**

This study included patients aged 65 years and older that were treated at one Level 1 trauma center between 2007 and 2018. Inclusion criteria: Proximal humerus fracture treated with or without allograft, conclusive data-sets. Exclusion criteria: prior surgical treatment of the proximal humerus, open fracture with bone loss, neurological damage. Patients were stratified according to the use of allograft augmentation in two groups: Group NA (no allograft augmented PHILOS) and Group A (PHILOS with allograft augmentation). Comorbidity was assessed using the Charlson Comorbidity Index (CCI). Fractures were graded according to the classification by Neer. Radiographic union was analyzed at 6 weeks, 12 weeks, and at year follow up. Complications include surgical site infection, implant failure, humeral head necrosis, or delayed union. Allograft was used in cases of 1inch/3cm^3^ bone-loss or an egg-shell situation, where the patient refused arthroplasty.

**Results:**

This study included 167 patients, with 143 (85%) in the Group NA, and 24 (15%) in the Group A. There were no significant differences in age, gender, injury distribution, and distribution of Neer classification or CCI. Patients in Group A had significantly lower T-scores preoperatively (-2.87 ± 1.08 versus -0.9 ± 2.12, p = 0.003). No difference occurred in any of the complications. At one-year follow-up, the range of motion was comparable in both groups.

**Conclusion:**

In patients with allograft augmentation and severe osteopenia, similar clinical and radiological results were obtained when compared with patients with better preoperative bone density scores (T-scores, DEXA). In view of a lack of guidelines indicating the indication for the use of allograft, this difference may be worth further study.

## Introduction

The increase in life expectancy, and the increase in active life-style leads projections for 2050 to indicate that more than 20.1% of the population will be 65 years and older [1]. One consequence is the increase in elderly orthopedic trauma patients. In elderly patients, proximal humerus fractures are the third most common fracture pattern [2]. The incidence of proximal humerus fractures in the geriatric population has increased by 28% between 1990 and 2010 [3]. One of the most important challenges in the surgical treatment of proximal humerus fractures in the elderly is the poor bone quality. Fractures of the proximal humerus are among the most common osteoporotic fractures of long bones [4]. Impaired bone quality increases the risk of screw penetration, delayed union, or instability [5]. This may occur despite the use of locked plating [6]. The Proximal Humeral Internal Locking System (PHILOS) plate represents one special entity of locked plating. It has been used successfully for the treatment of proximal humerus fractures [7]. However, a recent study showed that 18% of patients over 65 years of age had complications that needed secondary intervention following their index surgery [7]. Another study showed that 22% of patients treated with PHILOS had to undergo a second intervention [8]. Out of all these patients, 30.8% had either loss of reduction (3.8%), screw perforation (19.2%), or non-union (7.7%) [8]. Cement augmented fixation has been studied and appears to have biomechanical advantages [9], as did biologic or synthetic bone substitutes [4]. Demineralized bone matrix has been used in several orthopedic trauma conditions such as in the treatment of critical size defects [10]. Allografts have also been used in several studies [11] but indication in the treatment of acute proximal humerus fractures in the elderly remains controversial. Therefore, the aim of this study was to answer the following questions regarding allografts in the surgical treatment of proximal humerus fractures in the elderly:

1. Can allograft augmentation influence functional and clinical outcome in unstable proximal humerus fractures?
2. Can a radiographic examination or preoperative DEXA measurement be helpful in decision making for the use of allograft augmentation?

## Methods

This study was conducted with the approval of the regional ethical committee (Kantonale Ethikkommission, KEK, Zürich) and the institutional review board (IRB, no 2019–01957 and 2018–00146).

### Study design, study population, and definitions

This retrospective cohort study included patients, aged 65 years and older, that were treated at one Level 1 trauma center. All eligible patients suffered a proximal humerus fracture that was treated with PHILOS between 2007 and 2018. Inclusion criteria for this study were: proximal humerus fracture, classified by Neer [12], treated with PHILOS, leading surgeon FA, and a conclusive data-set.

Exclusion criteria were prior surgical intervention of the proximal humerus, open fracture with bone loss, neurological damage, genetic disorders affecting the musculo-skeletal system, or patients that did not attend at least one follow up. The follow-up included clinical and radiologic assessments 6 weeks, 12 weeks, and one year after surgery. These follow-up periods were based on routine clinical practice. The clinic management contacted patients who did not attend the follow up. Reasons for missing follow-up included deceased patients and the wish of the patient to attend follow-up with a physician near their hometown. Injury distributions were stratified according to the number and anatomic location of the injuries. Multiple injury was defined according to the “Berlin definition” [13]. Patients’ comorbidity was assessed using the Charlson Comorbidity Index (CCI) based on information given on admission and in consulting reports [14]. Patients were further assessed for osteoporosis. They were grouped, according to the maximum information provided at discharge or at the end of follow-up, into osteoporotic patients with and without pharmaceutical treatment. T-scores were calculated based on radiographic measurements with dual-energy x-ray absorptiometry (DEXA) at the lumbal vertebrae prior to surgery. A T-score below -2.5, defined osteoporosis; a T-score between -1.5 and -2.5 defined osteopenia [15]. “Osteoporosis with medication” indicates the need for bisphosphonates or other specific medications targeted at treating osteoporosis. According to our in-hospital guidelines, all patients with fragility fractures received vitamin D and calcium supplementation.

Patients were stratified according to the use of allograft augmented PHILOS in two groups: Group NA (no allograft augmented PHILOS) and Group A (PHILOS with allograft augmentation).

### Allograft

We used commercially available cancellous bone allograft (Arcuate^™^, Cancellous Bone Block, 17mm x 20mm x 30mm, Medtronic, Memphis, Tennessee, USA). This allograft was tapered according to the need of the given defect. The indication for surgical treatment of proximal humerus fractures were the patient`s wish for immediate exercise stability and the pain-free aftercare. This study is a series performed as a single surgeon experience. The senior author has performed all cases as leading surgeon (FA). For the allograft enhancement in proximal humerus fractures, the threshold level was set at a minimum of 1inch/3cm^3^ bone-loss, or an eggshell situation where the patient refuses an arthroplasty, according to our in-hospital guidelines. We selected a threshold of approximately 1inch/3cm^3^ because we had seen failures in previous surgeries, when a screw did not capture smaller bone grafts. We are aware that this might be a possible drawback but have not seen a single failure after this rule was applied.

### Outcome measures

Complications include deep surgical site infection that required intervention (either secondary surgery or antibiotic treatment). Further, avascular humeral head osteonecrosis that was diagnosed after acute onset of shoulder pain without clear inciting event, loss of motion, crepitus and radiographic osteolytic lesion defined aseptic osteonecrosis of the humerus head [16]. Delayed union was defined as visible fracture line without bridging callus at one year follow-up. Radiological union was defined when the junction was no longer visible and a bridged callus was visible on three of four cortices [17].

The functional outcome included the range of motion at one year follow up as well as the Disability of Arm, Shoulder and Hand (DASH) score [18].

The assessment of pain was based on the need of regular pain medication, based on the pain of the shoulder. At each visit, patients were asked whether they needed analgesics or whether they were pain free.

### Quality of fracture reduction

Malreduction was defined by meeting at least one of the three criteria defined by Schnetzke et al [19]: medial head shaft displacement greater than 5 mm, greater tuberosity cranialization of more than 5 mm, and neck shaft angle greater than 150° or major varus displacement. Acceptable reduction was achieved after a head-shaft displacement of less than 5 mm, head-shaft alignment between 110° and 120° or minor varus displacement, or cranialization of the greater tuberosity of less than 5 mm. These parameters for fracture reduction were based on the definition for quality of fracture reduction by Schnetzke et al [19].

Loss of reduction was assessed at the first postoperative standardized x-ray, in anterior-posterior view as well as in Neer-view, and on all follow up x-rays. Loss of reduction was documented, as it included fracture collapse, implant failure, or screw penetration. The need of secondary surgical intervention led to the exclusion of the patient.

### Statistical analysis

Continuous variables are displayed with mean and standard deviation. Categorical variables are presented with number and percentage. Group comparisons of continuous variables have been performed using a students t-test; comparisons of categorical variables used the chi-squared test. Group comparisons of more than two groups were performed using ANOVA. All analyses have been performed using R (R Core Team (2018). R: A language and environment for statistical computing. R Foundation for Statistical Computing, Vienna, Austria. URL https://www.R-project.org/)

## Results

**Fig 1:**
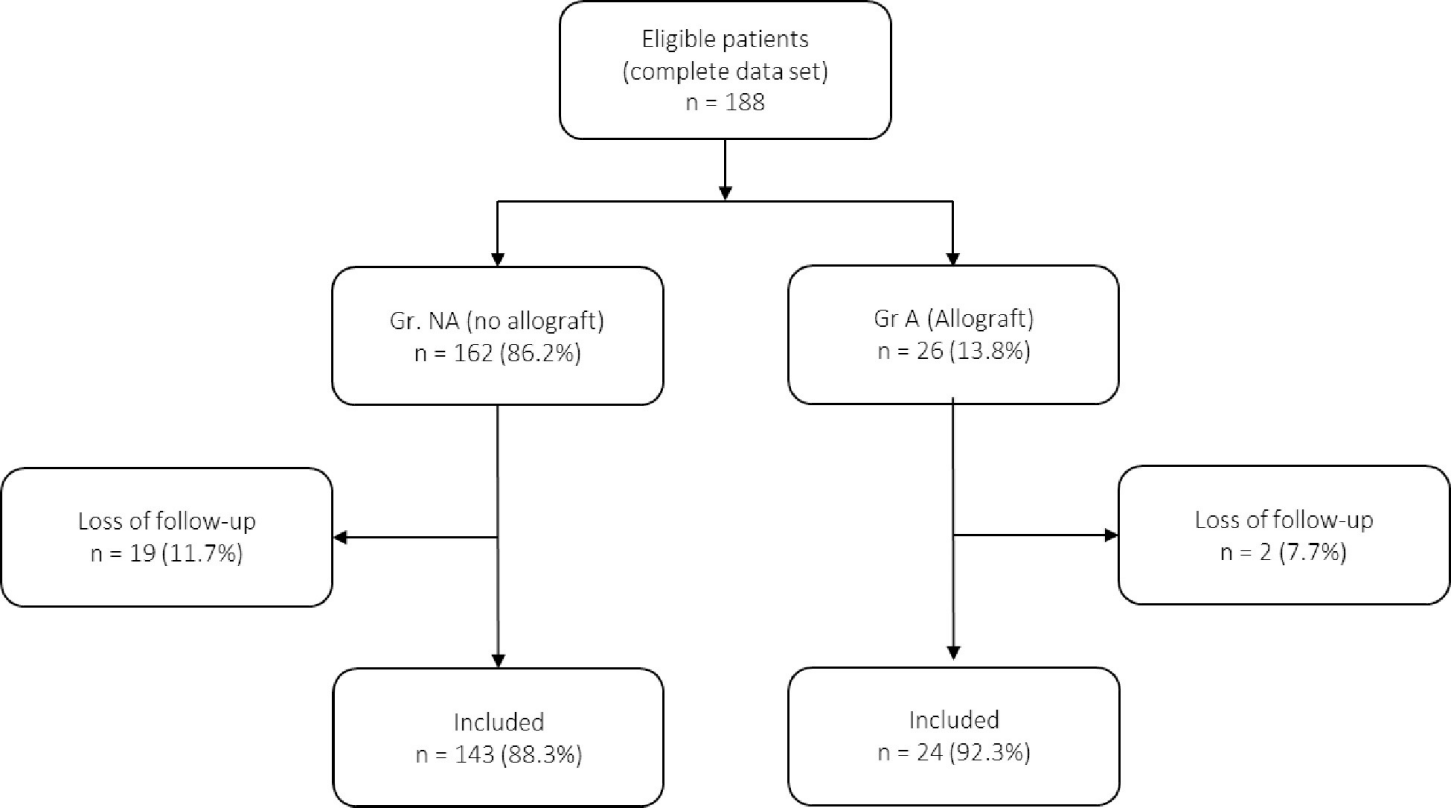
Flow chart of eligible patients and treatment arms. Eligible patients met the inclusion criteria of surgical treatment of proximal humerus fractures with Proximal Humeral Internal Locking System (PHILOS). Reasons for loss of follow-up include incomplete follow-up data, or patients who had follow-up visits at other institutions or with their general physicians.

The data analysis revealed 188 eligible patients. Of these, 21 (11.2%) patients did not fulfill the inclusion criteria, or had follow-up data, and 167 patients (88.8%) were therefore included for analysis (Fig 1). Group A included 24 (14.4%) patients and Group NA 143 (85.6%) patients. These groups were comparable in age, gender distribution and injury severity or distribution. Patients in both groups had similar demographics, comparable injury distribution, and similar length of stay (Table 1). Further, distribution of fracture classifications, comorbidities and severity of comorbidities were comparable in both groups. However, our data show that patients in Group A had significantly lower T- scores (-2.87 ± 1.08) compared to patients in Group NA (-0.9 ± 2.12, p = .003) (Table 2).

**Table 1:** Patient demographics.

	Group NA	Group A	p
n	143	24	
Age at injury [years], mean (SD)	77.71 (8.16)	77.34 (8.02)	n.s.
Female n (%)	110 (76.9)	20 (83.3)	n.s.
Injury distribution, n (%)			n.s.
Isolated proximal humerus fracture n(%)	113 (79.0)	20 (83.3)	n.s.
Prox. Humerus fracture and additional Injury except ipsilateral upper extremity	15 (10.5)	2 (8.3)	n.s.
Prox. Humerus fracture and additional Injury of the ipsilateral upper extremity	8 (5.6)	0 (0.0)	n.s.
Multiple other injuries (polytrauma, ISS 16 and above)	7 (4.9)	2 (8.3)	n.s.
Length of stay [days], mean (SD)	10.34 (9.84)	11.21 (6.60)	n.s.

Group NA = No allograft augmented PHILOS

Group A = Allograft augmented PHILOS

SD = Standard Deviation

n.s. = not significant (p>.05)

**Table 2:** Fracture classification and comorbidities.

	Group NA	Group A	p
Neer Classification n (%)			n.s.
Type 1	111 (79.9)	16 (66.7)	n.s.
Type 2	19 (13.7)	6 (25.0)	n.s.
Type 3	6 (4.3)	2 (8.3)	n.s.
Type 4	3 (2.2)	0 (0.0)	n.s.
Charlson Comorbidity Index (CCI) [points], mean (SD)	4.45 (2.05)	5.17 (2.71)	n.s.
Bone quality according T-score and medication, n (%)			n.s.
No Osteoporosis	12 (21.1)	3 (27.3)	n.s.
Osteopenia	13 (22.8)	4 (36.4)	n.s.
Osteoporosis (no medication at time of injury)	21 (36.8)	3 (27.3)	n.s.
Osteoporosis with medication	11 (19.3)	1 (9.1)	n.s.
T-score (mean (SD))	-0.90 (2.12)	-2.87 (1.08)	.003

Group NA = No allograft augmented PHILOS

Group A = Allograft augmented PHILOS

SD = Standard Deviation

n.s. = not significant (p>.05)

Comparing the quality of reduction in both groups, our data show no significant differences in the quality of reduction immediately after surgery or at the end of follow up (p = .098, resp. p = .322) as presented in Table 3.

**Table 3:** Quality of reduction according to Schnetzke et al [19].

	Group NA	Group A	p
n	143	24	
Quality of reduction postoperatively n (%)			n.s.
Anatomical reduction	93 (65.5)	10 (43.5)	n.s.
Acceptable reduction	42 (29.6)	12 (52.2)	n.s.
Malreduction	7 (4.9)	1 (4.3)	n.s.
Quality of reduction end of follow up n (%)			n.s.
Anatomical reduction	72 (54.1)	9 (39.1)	n.s.
Acceptable reduction	38 (28.6)	10 (43.5)	n.s.
Malreduction	23 (17.3)	4 (17.4)	n.s.

Group NA = No allograft augmented PHILOS

Group A = Allograft augmented PHILOS

n.s. = not significant (p>.05)

Mean surgery time in Group A was 162.1 ± 69.2 min, whereas surgery time in Group NA was 116.6 ± 46.3 min. Despite the significant difference in surgery time in the groups (p < .001), the rate of complications were comparable in both groups (Table 4).

**Table 4:** Complications during follow-up.

	Group NA	Group A	p-value
Deep surgical site infection during hospitalization, n(%)	1 (0.7)	1 (4.2)	n.s.
Radiological loss of reduction within 6 weeks, n(%)	12 (8.7)	3 (13.0)	n.s.
Revision surgery due to implant failures within 12 weeks, n(%)	2 (1.4)	0 (0.0)	n.s.
Screw penetration	10 (7.2)	4 (17.4)	n.s.
Fracture collapse	10 (7.2)	2 (8.7)	n.s.
Delayed Union	30 (22.2)	1 (4.2)	n.s.
Humeral head necrosis	14 (10.4)	1 (4.2)	n.s.

Group NA = No allograft augmented PHILOS

Group A = Allograft augmented PHILOS

n.s. = not significant (p>.05)

The range of motion was measured as a mean of 8 ± 2 months. Both groups were comparable in range of motion and DASH score. Despite no statistical significance, Group A showed a slightly increased range of retroversion, adduction, and external rotation. Group NA had higher values in flexion, abduction, and internal rotation. The differences in range of motion are not statistically significant except the differences in internal rotation (Table 5). However, comparing pain, significantly more patients in Group A had pain 6 weeks after surgery compared to Group NA (p = .047) (Table 5).

**Table 5:** Functional outcome after one year follow-up and rate of shoulder pain during the follow-up period.

	Group NA	Group A	p-value
Follow-up time [months], mean (SD)	8.18 (4.57)	8.37 (4.21)	n.s.
Retroversion [°], mean (SD)	35.92 (19.17)	40.77 (50.61)	n.s.
Flexion [°], mean (SD)	115.88 (44.02)	112.22 (37.97)	n.s.
Abduction [°], mean (SD)	111.12 (45.99)	97.50 (34.93)	n.s.
Adduction [°], mean (SD)	30.67 (12.02)	34.09 (9.44)	n.s.
External Rotation [°], mean (SD)	48.01 (23.00)	49.71 (28.48)	n.s.
Internal Rotation [°], mean (SD)	71.08 (19.44)	59.69 (25.98)	n.s.
DASH-score after 12 months [points], mean (SD)	14.28 (15.25)	11.21 (NA)	NA
Pain level, n (%)			
6 weeks post op (%)	104 (75.9)	21 (91.3)	.047
3months post op (%)	47 (35.6)	12 (52.2)	n.s.
12months post op (%)	17 (13.3)	3 (13.6)	n.s.

Group NA = No allograft augmented PHILOS

Group A = Allograft augmented PHILOS

SD = Standard Deviation

n.s. = not significant (p>.05)

Post op = post operatively

## Discussion

Poor bone quality in the elderly is one of the challenges during the operative fixation using PHILOS plate. Studies indicate a positive effect on stability after the use of autografts, allografts, or cement augmentation in osteoporotic bone [20, 21]. None of the studies described clear indications for the use of allograft. Our study revealed the following results:

1. We did not observe a difference in the complication rate, or regarding long term clinical and radiological outcome between both groups
2. Group A had a significantly lower T-score compared to Group NA prior to surgery

It has been shown that the use of allograft improves stability of the reduced fracture [20] that facilitates fracture healing. Alongside the importance of stability, an appropriate biochemical environment is essential for fracture healing. Allograft bone has been shown to have properties that support bone formation [21]. Further, the allograft bone serves as a scaffold to support bone healing, and even remodelling [22]. Allograft augmented constructs have been shown to be stiffer and demonstrate less migration of the humeral head fragments [23]. Based on these properties and our finding, allograft augmentation serves as a useful augmentation technique for proximal humerus fractures in elderly patients treated with PHILOS.

One might argue however, that bone augmentation might increase complication rates based on increased operation time, potentially increased blood loss, or due to the more invasive reduction procedure required to insert allograft bone at the required site [24]. Our data indicate significantly higher operation times when allografts are used compared to PHILOS without allograft augmentation. However, complications rates in both groups were comparable. These results are similar to recently published studies comparing outcome after augmentation with fibular allografts [22–24]. Given the generally increased complication rate of locking-plate fixation of proximal humerus fractures in the elderly [25], allograft augmentation might be one adjunct to improve stability.

Some authors suggest that the use of allograft is associated with comparable or improved functional outcome [26]. Locking plate osteosynthesis of the proximal humerus fracture demonstrated good results in terms of early functional training [27] and supports stability and facilitates early motion and functional training [24].

### Indications for the use of allograft

This study showed significantly different T-scores, with Group A having the lower T-score compared to Group NA. This result might indicate the T-score to serve as guidance for allograft augmentation. Several studies investigated potential benefits of allograft augmentation (Addendum). However, none of the studies investigated a clear indication for the use of allograft [20, 28]. One study described, “If the reduction was difficult and was considered less likely to be maintained because of weak bone quality or comminution, a strut allograft was used” without further clarifications [29]. A further study compared in a retrospective design surgical failure after the use of fibular allograft without mentioning indications or guidelines for the use of allograft [30]. Out of the recently published studies investigating the effects of allograft in proximal humerus fractures, only two articles analysed preoperative T-scores. One of them did not perform group comparisons [22] and the second study did not focus on the value of T-scores [30]. The current study is the first that investigated potential indications for allograft augmentation during PHILOS and found significantly different T-scores as potential indicators for treatment options.

#### Strengths and limitation

The lack of patients with Neer Type 4 fracture in the allograft group might introduce a bias. However, there are still no clear indications for the use of allograft. Since one of our main result reveals a significant difference in pre-operative T-score (DEXA), this result may outweigh the risk of a selection bias in view of comparable clinical and radiological results. Also, a prospective randomized controlled trials would be preferable to provide definitive recommendations for augmenting PHILOS with allograft in the elderly.

We feel that our pain assessment based on the need of analgesics is an advantage. The numeric analogue scale (NAS) for measuring pain level is not routinely documented during the follow-up visit, but we still feel that the need of analgesics is an adequate measure of pain. Furthermore, exploratory analysis show significantly lower DEXA scores in Group A. DEXA is not routinely measured in our department and this difference might, therefore, be underpowered.

## Conclusion

The use of allograft-augmented locked plating in elderly patients with osteopenia is comparable with non augmented locked plating in patients with less osteopenic bone. It may be worth to investigate in larger trials whether the T-score could be an indicator for allograft augmentation in geriatric proximal humerus fractures.

## Supporting information

S1 Data (DOCX)
