# Supplementary material for: Treatment of proximal humerus fractures in geriatric patients - Can pathological DEXA results help to guide the indication for allograft augmentation?
Source: PLoS One. 2020 Apr 9;15(4):e0230789. doi: 10.1371/journal.pone.0230789 (PMC7145012; doi:10.1371/journal.pone.0230789)
Supplement: S1 Data — (DOCX) [file pone.0230789.s001.docx]

| Addendum: Overview of recent literature based on searches in Medline, Embase, Web of Science, and Cochrane Database | | | | | | | | |
| --- | --- | --- | --- | --- | --- | --- | --- | --- |
| Author | Year | Journal | Title | Comparator | Number of Patients | Measurement of radiological healing | DEXA measurement | Main Outcome |
| Lee et al. | 2019 | Bone Joint J | Outcomes of locking plate fixation with fibular allograft augmentation for proximal humeral fractures in osteoporotic patients | Fibular strut allograft (LP) versus fibular allograft (FA) | Group LP n=52 Group FA n=45 | Humeral neck-shaft angle Humeral Head height  immediately after surgery | No | Fibular strut graft with satisfactory short-term clinical and radiological outcome |
| Chen et al. | 2016 | Orthop. Traumatol.-Surg. Res. | Comparison of Intramedullary fibular Allograft With Locking compression Plate Versus Shoulder Hemi-Arthroplasty for Repair of Osteoporotic Four-Part Proximal Humerus Fracture: Consecutive, Prospective, Controlled, and Comparative Study | Fibular allograft (IFA) versus Hemiarthroplasty (HA) | Group IFA n=30 Group HA n=30 | Time to union and radiological complications of fracture not further sepcified | No | IFA and HA comparable radiological and functional |
| Zhao et al. | 2019 | Orthop. Surg. | Comparison of the Effects of Proximal Humeral Internal Locking System (PHILOS) Alone and PHILOS Combined with Fibular Allograft in the Treatment of Neer Three- or Four-part Proximal Humerus Fractures in the Elderly | PHILOS (control) versus PHILOS + fibular allograft (observation) | Control n=21 Observation n=12 | Neck-shaft angle Humeral Head Heigth | No | Group observation with better functional outcome, lower complication rate |
| Davids et al. | 2019 | J Orthop Trauma | Comparison of Locked Plating of Varus Displaced Proximal Humeral Fractures With and Without Fibula Allograft Augmentation | PHILOS (no Allograft) versus PHILOS + fibular allograft (Allograft | Group No Allograft n=75 Group Allograft n=27 | Failure, Shift, not further specified | No | Limited value in using fibula allograft (adds time, cost and risk) |
| Chen et al. | 2018 | Biomed Res. Int. | The Augment of the Stability in Locking Compression Plate with Intramedullary Fibular Allograft for Proximal Humerus Fractures in Elderly People | LCP (Group I) versus LCP with fibular allograft (Group II) | Group I n=42 Group II n=47 | Humeral neck-shaft angle Humeral Head height | No | LCP with fibular allograft with better functional outcome and lower complication rate |
| Sanchez-Sotelo et al. | 2017 | J. Bone Joint Surg.-Am. Vol. | Allograft-Prosthetic Composite Reconstruction for Massive Proximal Humeral Bone Loss in Reverse Shoulder Arthroplasty | Primary versus Revision Reverse Allograft-prosthesis composites (APCS) | Primary APC n=8 Revision APC n=18 | Radiological union when junction no longer visible bridged callus on 3 of 4 cortices | No | Structural allograft reconstruction with durable outcome and acceptable complication rate |
| Cha et al. | 2017 | J. Shoulder Elbow Surg | Treatment of Comminuted Proximal Humeral Fractures Using Locking Plate With Strut Allograft | LCP versus LCP with strut allograft | LCP n=32 LCP with strut allograft n=20 | Neck-shaft angle Humeral Head Heigth | No | LCP and LCP with strut allograft comparable |
| Chen et al. | 2015 | J. Orthop. Surg. Res | Clinical outcomes of allograft with locking compression plates for elderly four-part proximal humerus fractures | Anatomical allograft versus Fibula shaft allograft | Anatomical n=7 Fibular Shaft n=15 | Avascular necrosis, varus displacement of humeral head, screw penetration | Yes (no group comparison) | Fibular shaft allograft comparable to anatomical allograft in functional outcome |
| Hinds et al. | 2015 | J. Shoulder Elbow Surg | Geriatric proximal humeral fracture patients show similar clinical outcomes to non-geriatric patients after osteosynthesis with endosteal fibular strut allograft augmentation | Geriatric versus non-geriatric patients | Geriatric n=34 Non-geriatric n=37 | Humeral head heigth Loss of reducation Screw cut out Osteonecrosis | No | Both groups with comparable clinical outcome |
| Zhu et al. | 2014 | Chin. Med. J | Locking plate fixation combined with iliac crest bone autologous graft for proximal humerus comminuted fracture | LCP+autologeous crest bone graft versus LCP | LCP+ n=18 LCP n=22 | Humeral head heigth Osteonecrosis, Screw penetration, nonunion Radiographic union | NA (Abstract only) | LCP+iliac crest as aneffective technique |
| Kim et al. | 2018 | J. Shoulder Elbow Surg | Which additional augmented fixation procedure decreases surgical failure after proximal humeral fracture with medial comminution: fibular allograft or inferomedial screws? | Fibular Allograft (FA) versus additional inferomedial screws (IMS) | FA n=84 IMS n=80 | Radiological union with bone bridging across both cortices, non union as the absence of progression of union | Yes (similar in both groups) | FA clinically and radiologically superior to IMS. |
